# A new type of vanadium carbide V_5_C_3_ and its hardening by tuning Fermi energy

**DOI:** 10.1038/srep21794

**Published:** 2016-03-01

**Authors:** Wandong Xing, Fanyan Meng, Rong Yu

**Affiliations:** 1 Department of Physics, University of Science and Technology Beijing, Beijing 100083, China; 2 National Center for Electron Microscopy in Beijing, School of Materials Science and Engineering, Tsinghua University, Beijing 100084, China

## Abstract

Transition metal compounds usually have various stoichiometries and crystal structures due to the coexistence of metallic, covalent, and ionic bonds in them. This flexibility provides a lot of candidates for materials design. Taking the V-C binary system as an example, here we report the first-principles prediction of a new type of vanadium carbide, V_5_C_3_, which has an unprecedented stoichiometry in the V-C system, and is energetically and mechanically stable. The material is abnormally much harder than neighboring compounds in the V-C phase diagram, and can be further hardened by tuning the Fermi energy.

Transition metal carbides have attracted continuing interest due to their excellent physical properties and wide engineering applications^1^,^2^,^3^,^4^,^5^,^6^. Because of the coexistence of the covalent, ionic, and metallic bonding types between the transition metals and carbon, the transition metal carbides usually have various stoichiometries. The flexibility in stoichiometry leads to rich chemical and physical behaviors, and provides a lot of candidates for materials design.

The V-C system is a typical binary system which has many different stoichiometries. V_2_C, V_4_C_3_, V_6_C_5_, V_8_C_7_, and VC have been synthesized and investigated for many years^7^,^8^,^9^,^10^,^11^. T_5_M_3_ is a common stoichiometry composed of transition metals T and main-group elements M. There are several structure types for this specific stoichiometry, including *D*8_8_ (Mn_5_Si_3_, hexagonal, P6_3_/mcm, No.193), *D*8_*l*_ (Cr_5_B_3_, tetragonal, I4/mcm, No.140), and *D*8_*m*_ (W_5_Si_3_, tetragonal, I4/mcm, No.140), with their prototypes and space groups given in the parentheses. For silicides of group VB transition metals, both Ta_5_Si_3_ and Nb_5_Si_3_ have the Cr_5_B_3_-type structure, and V_5_Si_3_ has the Mn_5_Si_3_-type structure^12^,^13^,^14^,^15^,^16^,^17^. But carbides with this stoichiometry have never been synthesized nor theoretically studied.

In this work, we take the V-C system as a model system to explore the possibility to design new materials by changing the stoichiometry. The calculations were performed to investigate the crystal structure, phase stability, electronic structure, and mechanical properties of V_5_C_3_. The results show that the Cr_5_B_3_-type V_5_C_3_ is stable mechanically, dynamically, and thermodynamically, and can be synthesized at high pressures. The hardness of the hard material can be enhanced further through tuning the Fermi energy.

## Results and Discussion

**Figure 1:**
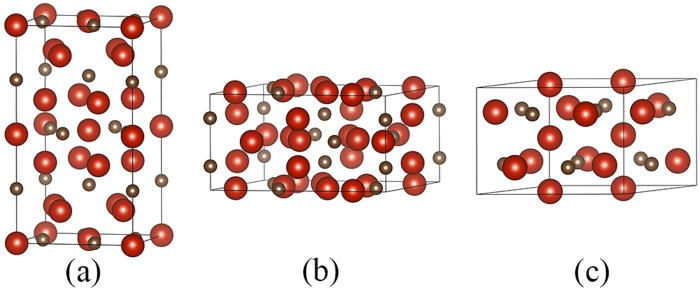
Structure models of V_5_C_3_: (**a**) Cr_5_B_3_-type, (**b**) W_5_Si_3_-type, (**c**) Mn_5_Si_3_-type. The large and small spheres represent V and C, respectively.

As mentioned above, three typical structure types for T_5_M_3_, i.e., Mn_5_Si_3_, Cr_5_B_3_, and W_5_Si_3_ types are considered in this work, as shown in Fig. 1. For comparison, the known vanadium carbides in the V-C phase diagram, i.e. VC (cubic, *Fm-3m*), V_2_C (orthorhombic, *Pbcn*), V_4_C_3_ (hexagonal, *R-3m*), V_6_C_5_ (hexagonal, *P3*_*1*_), and V_8_C_7_ (cubic, *P4*_*3*_*32*) are also included in the calculations.

The formation enthalpy was calculated using the following equation,


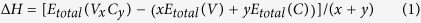


where *E*_*total*_(*V*_*x*_*C*_*y*_) was the obtained total energies for the considered vanadium carbide, *E*_*total*_(*V*) and *E*_*total*_(*C*) were the total energy of the pure metal V and the graphite, respectively. The calculated lattice parameters and formation enthalpy ∆*H* at zero pressure are listed in Table 1. For the known vanadium carbides, the calculated values are in good agreement with previous calculation values.

**Figure 2:**
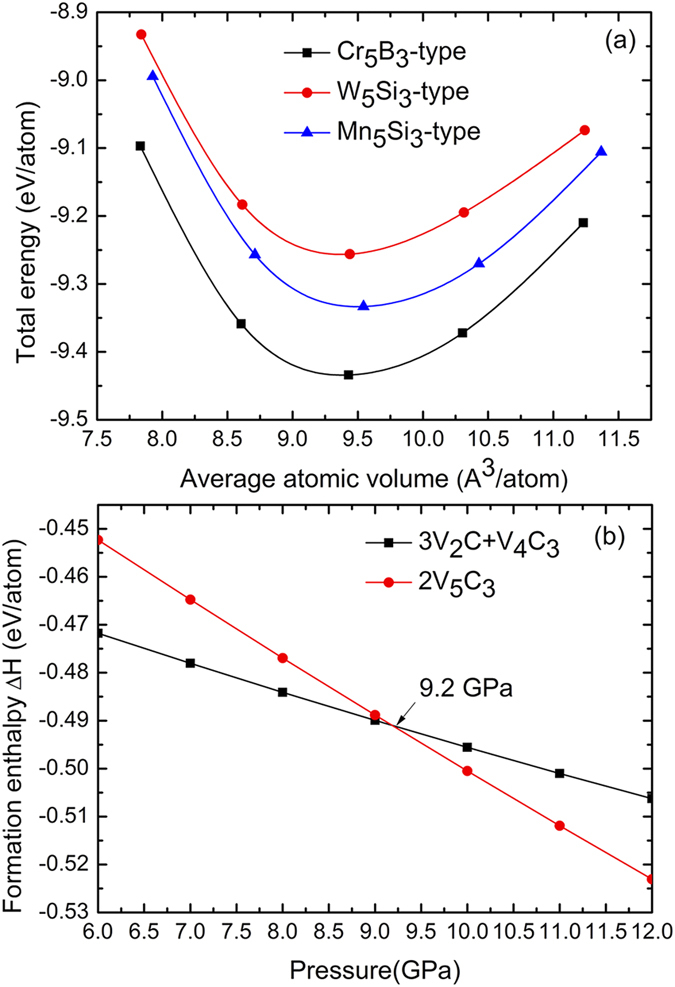
(**a**) Energy-volume relationships for the Cr_5_B_3_-type, W_5_Si_3_-type and Mn_5_Si_3_-type V_5_C_3_. (**b**) The relative enthalpy-pressure diagram of the Cr_5_B_3_-type V_5_C_3_ and its respective competing phases.

The total energies of V_5_C_3_ as a function of volume for the three structure types are plotted in Fig. 2(a). The Cr_5_B_3_-type V_5_C_3_ has the lowest energy at all the volumes. Hereafter, only the Cr_5_B_3_-type V_5_C_3_ is considered unless stated otherwise. It is worth noting that the formation enthalpies of these vanadium carbides are all negative at zero pressure. The negative formation enthalpies indicate that the carbides are more stable than the mixture of elemental V and C.

For a compound to be synthesized experimentally, it is more reliable to compare its enthalpy with the known compounds of neighboring stoichiometries. In the V-C phase diagram, V_5_C_3_ would locate in the two-phase region bounded by V_2_C and V_4_C_3_. Therefore, we need to compare the formation enthalpy of V_5_C_3_ with the mixture of V_2_C and V_4_C_3_. The formation enthalpies as a function of pressure have been calculated for both V_5_C_3_ and the mixture of V_2_C and V_4_C_3_, as shown in Fig. 2(b). The mixture is more stable than V_5_C_3_ under pressures below 9.2 GPa, above which V_5_C_3_ becomes more stable. It indicates that V_5_C_3_ is thermodynamically more stable than that of the mixture at high pressures.

The elastic properties of a material are very important as they determine the mechanical stability, strength, hardness, and ductile or brittleness behavior. The calculated elastic constants *C*_*ij*_, the minimum elastic eigenvalue *λ*_1_ 18, bulk modulus *B*, shear modulus *G*, Young’s modulus *E*, Poisson’s ratio *ν* and hardness *H*_*ν*_ of these vanadium carbides are listed in Table 2. The calculated values of V_2_C, V_4_C_3_, V_6_C_5_, V_8_C_7_, and VC in this work are in good agreement with the previous calculation values.

The Cr_5_B_3_-type V_5_C_3_ is tetragonal. For a tetragonal system, the mechanical stability criteria are given by *C*_11_ > 0, *C*_33_ > 0, *C*_44_ > 0, *C*_66_ > 0, *C*_11_ − *C*_12_ > 0, *C*_11_ + *C*_33_ − 2 *C*_13_ > 0, and 2(*C*_11_ + *C*_12_) + *C*_33_ + 4 *C*_13_ > 0^19^. The elastic constants of the Cr_5_B_3_-type V_5_C_3_ satisfy these stability conditions, indicating that it is mechanically stable.

**Figure 3:**
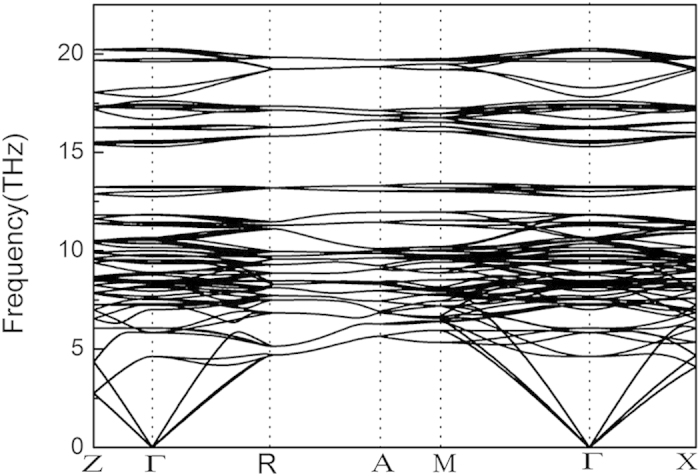
Phonon dispersions of the Cr_5_B_3_-type V_5_C_3_ at zero pressure along high symmetry directions of the Brillouin zone.

The phonon dispersions were calculated to verify the dynamical stability of the Cr_5_B_3_-type V_5_C_3_. A dynamically stable crystal structure requires that all phonon frequencies should be positive^20^. As shown in Fig. 3 for the Cr_5_B_3_-type V_5_C_3_ at zero pressure, it is clear that no imaginary phonon frequency can be found in the whole Brillouin zone, indicating that the Cr_5_B_3_-type V_5_C_3_ is dynamically stable under ambient conditions.

**Figure 4:**
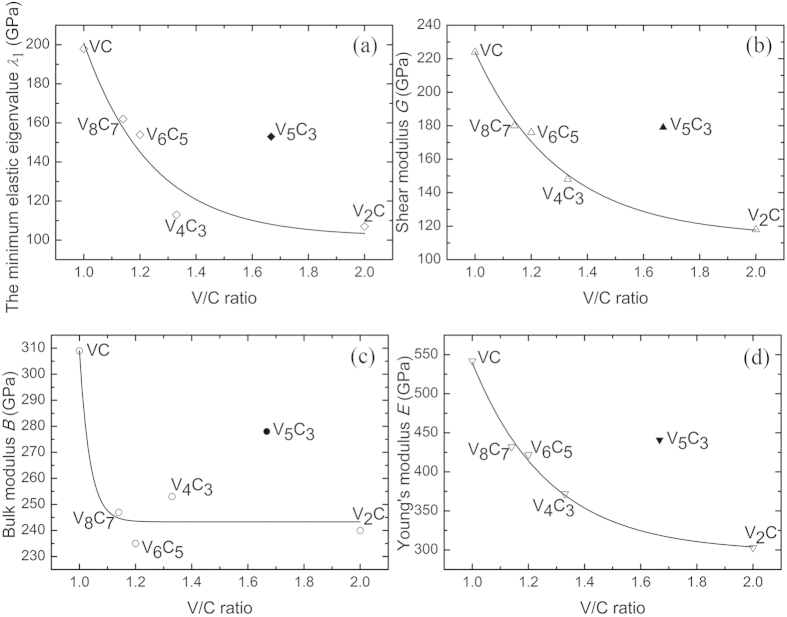
(**a**) The minimum elastic eigenvalue *λ*_1_, (**b**) shear modulus *G*, (**c**) bulk modulus *B* and (**d**) Young’s modulus *E* of vanadium carbides as a function of the V/C ratio. The lines are guide to the eye.

Because the hardness measurement involves complex deformation processes, including elastic deformations, plastic deformations, and fracture, it is difficult to obtain directly the hardness value of a material from first-principles calculations. Therefore, correlations between elastic moduli and hardness have been suggested as indirect indicators of materials hardness. A hard material should have a high bulk modulus to resist the volume contraction in response to an applied load, and a high shear modulus to resist shear deformation. Recently, the softest elastic mode has been shown to correlate better to the hardness number than the other elastic moduli^18^, indicating that elastic anisotropy is essential in determining the hardness. The elastic properties (*B*, *G*, *E*, and *λ*_1_) of V_5_C_3_ and the other previously known vanadium carbides as a function of the V/C ratio are plotted in Fig. 4. For the know vanadium carbides, the general trend is that the elastic moduli decrease with the V/C ratio. An abnormal increase occurs at V_5_C_3_, the elastic moduli of which are higher than both the neighboring V_2_C and V_4_C_3_.

**Figure 5:**
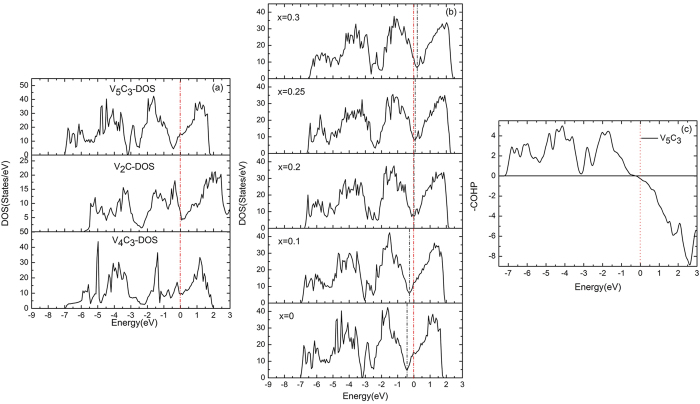
(**a**) Densities of states of V_5_C_3_, V_2_C and V_4_C_3_; (**b**) Densities of states; and (**c**) Crystal Orbital Hamilton Population (−COHP) analysis of V_5_C_3_. The red vertical dashed lines denote the Fermi level at zero and the black vertical dashed lines correspond to the energy valley.

In order to explain the origin of the stability and the abnormal mechanical properties of the Cr_5_B_3_-type V_5_C_3_, the electronic structure of V_5_C_3_, V_2_C and V_4_C_3_ has been analyzed. Their densities of states (DOS) are plotted in Fig. 5(a). They are metallic with non-zero DOS values at the Fermi level. There are valleys (sometimes called pseudogap) close to the Fermi level for all the three compounds. In general, the electronic states with lower energies than the valley are bonding orbitals, and those with higher energies are antibonding orbitals^21^. To clarify the nature of the chemical bonding near the Fermi level, we performed the Crystal Orbital Hamilton Population (−COHP) analysis^22^, which gives an idea about the participating orbital pair. The positive value represents the bonding states and negative value represents the antibonding states. As shown in Fig. 5(c) for V_5_C_3_, it is clear that the pseudogap separates the bonding and antibonding states appears. A deeper valley means that the bonding orbitals are more stabilized and the antibonding orbitals are more destabilized, forming strong chemical bonds. Among the three compounds, V_5_C_3_ has the deepest valley close to the Fermi level. Therefore, the stability and the abnormal mechanical properties of V_5_C_3_ can be attributed to the pseudogap effect^23^,^24^.

**Figure 6:**
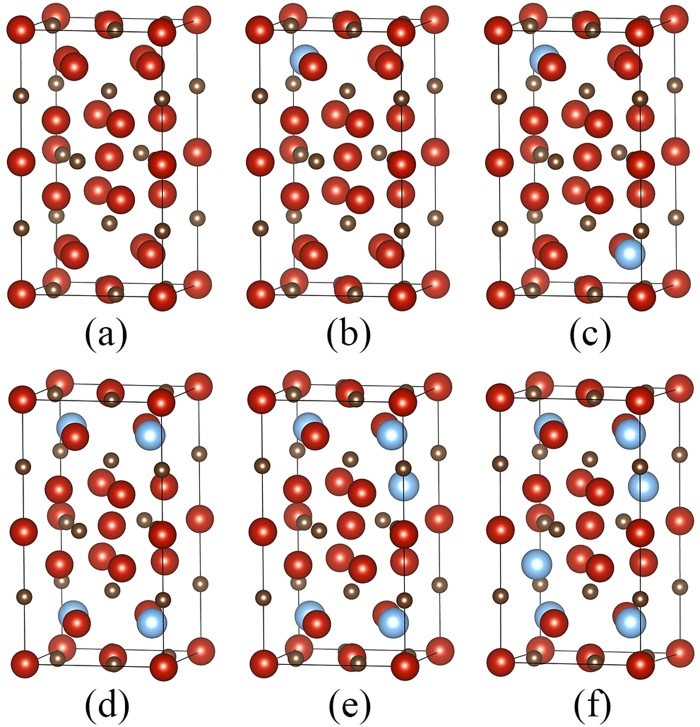
The supercells of (**a**) V_5_C_3_, (**b**) (V_0.95_Ti_0.05_)_5_C_3_, (**c**) (V_0.9_Ti_0.1_)_5_C_3_, (**d**) (V_0.8_Ti_0.2_)_5_C_3_, (**e**) (V_0.75_Ti_0.25_)_5_C_3_ and (**f**) (V_0.7_Ti_03_)_5_C_3_.

The electronic structure of V_5_C_3_ suggests an interesting method to improve its hardness. The Fermi level of V_5_C_3_ has a higher energy than the valley, indicating that some antibonding orbitals are occupied. Since the antibonding orbitals would weaken the chemical bonds, once they are made empty, the material could be further strengthened. We consider alloying V_5_C_3_ with Ti, which has one less valence electron than V. Since Ti is neighboring to V in the periodic table, it should be relatively easy to enter the lattice of V_5_C_3_. According to the rigid band model, the alloying element normally generates small changes in the nature of chemical bond in the host materials. The Cr_5_B_3_-type V_5_C_3_ with the alloying contents of 5 at.%, 10 at.%, 20 at.%, 25 at.%, and 30 at.% Ti were investigated. The supercells for the calculations are shown in Fig. 6. In order to minimize the interactions between the alloying atoms, they were placed as far as allowed in the supercells.

The DOS curves of V_5_C_3_ and its alloys (V_1−*x*_Ti_*x*_)_5_C_3_ were illustrated in Fig. 5(b). As expected, the Fermi level shifts to lower energies with increasing content of Ti from *x*_Ti_ = 0 to *x*_Ti_ = 0.3. The Fermi level is located at the valley for *x*_Ti_ = 0.2.

**Figure 7:**
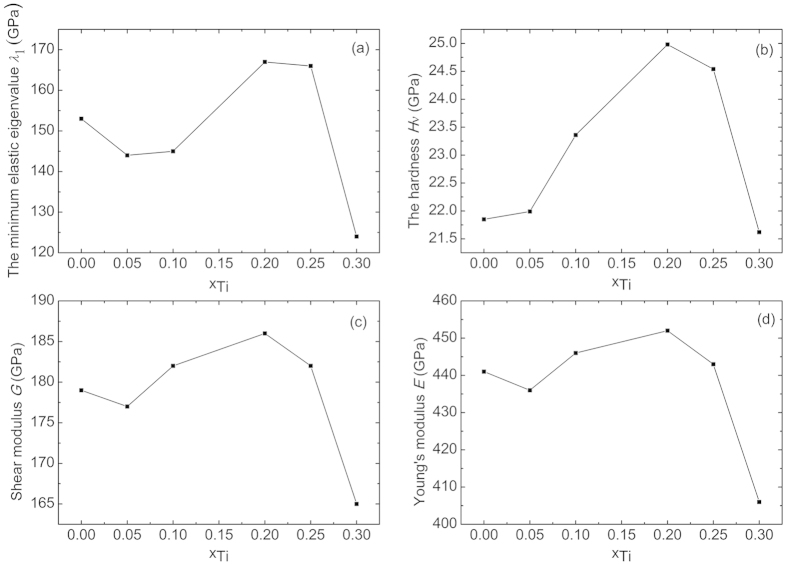
(**a**) The smallest elastic eigenvalue *λ*_1_, (**b**) hardness *H*_*ν*_, (**c**) shear modulus *G* and (**d**) Young’s modulus *E* of V_5_C_3_ alloys.

The calculated elastic constants are listed in Table 3. All the alloys are mechanically stable because the elastic constants of these alloys satisfy the mechanical stability criteria and there is no negative elastic eigenvalue. For the Cr_5_B_3_-type V_5_C_3_ and its alloys, the smallest elastic eigenvalue *λ*_1_ is *C*_66_, which represents the shear deformation in *xy* planes. The smallest elastic eigenvalue *λ*_1_, the hardness *H*_*ν*_, shear modulus *G* and Young’s modulus *E* are plotted in Fig. 7. A general trend is that *λ*_1_, *H*_*ν*_, *G* and *E* increase with the content of Ti from *x*_Ti_ = 0.05 to *x*_Ti_ = 0.2, where they reach their maxima, and then decrease as *x*_Ti_ increases further. The trend is exactly what we expect from the electronic structure analysis. At *x*_Ti_ = 0.2, the Fermi level is located at the valley in DOS. In this case, all of the bonding orbitals are occupied and the antibonding orbitals empty, leading to the strongest chemical bonds.

## Conclusions

In summary, the crystal structure, phase stability, electronic structure, and mechanical properties of V_5_C_3_ have been studied. It is demonstrated that the Cr_5_B_3_-type V_5_C_3_ is thermodynamically, mechanically, and dynamically stable, and can be synthesized under pressures above 9.2 GPa.

The elastic properties and electronic structures of (V_1−*x*_Ti_*x*_)_5_C_3_ alloys have also been investigated. When 20 at.% V is substituted by Ti, the Fermi level is tuned to the valley in DOS, giving the maximum hardness of V_5_C_3_ alloys. While V_5_C_3_ itself is not a superhard material, the electronic structure and the hardness optimization based on it suggest an interesting way for searching hard materials. The Fermi energy of a material can be tuned to maximize the occupation of bonding orbitals and minimize the occupation of antibonding orbitals, thus strengthening the material.

## Computational Methods

In this work, the density functional theory (DFT) calculations were performed using the projector-augmented wave (PAW) method^25^,^26^,^27^, as implemented in the Vienna Ab-initio Simulation Package (VASP) code^28^. The generalized gradient approximation (GGA)^29^ with the Perdew-Burke-Ernzerhof (PBE) scheme was used to describe the exchange-correlation function. Geometry optimization was carried out using the conjugate gradient algorithm. The plane-wave cutoff energy was 500 eV. The k-points were generated using the Monkhorst-Pack mesh^30^. Lattice parameters and atomic positions were optimized simultaneously. In order to obtain equilibrium volume of the materials, the total-energies were calculated at several fixed volume with the ionic positions and the cell shape allowed to vary. These total energies were then fitted with the Birch-Murnaghan equation of state^31^,^32^,^33^. The elastic constants were calculated using the universal-linear-independent coupling-strains (ULICS) method^34^, which is computationally efficient and has been widely used in calculations of single-crystal elastic constants^35^,^36^,^37^,^38^,^39^. Based on the single-crystal elastic constants, the bulk modulus *B* and the shear modulus *G* were calculated according to the Voigt-Reuss-Hill approximation^40^. Young’s modulus *E* and Poisson’s ratio *ν* were obtained by the following equation:


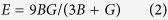



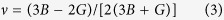


The hardness (*H*_*ν*_) of V_5_C_3_ is relative to *G* and *B* through the empirical formulabased on the Pugh modulus ratio *k* = *G*/*B*^41^,^42^:


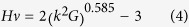


Phonon dispersions were calculated using the direct supercell method, as implemented in the PHONOPY code^43^,^44^. The Crystal Orbital Hamilton Population (−COHP) analysis have been performed to determine the bonding properties of the electronic states close to the Fermi level. Density functional method with LCAO basis sets, as implemented in the SIESTA code^45^, has been used to calculate the COHP. The PBE parameterization of GGA was used. The DZP basis sets were employed. The norm-conserving Troullier-Martins pseudopotentials^46^ were used for the core-valence interactions. The mesh cut-off value was set at 200 Rydberg and the Brillouin zone was sampled using Monkhorst-Packset of *k* points.

## Additional Information

**How to cite this article**: Xing, W. *et al.* A new type of vanadium carbide V_5_C_3_ and its hardening by tuning Fermi energy. *Sci. Rep.* **6**, 21794; doi: 10.1038/srep21794 (2016).

## Figures and Tables

**Table 1:** Calculated lattice parameters *a, b* and *c* (Å) and formation enthalpy ∆*H* (eV/atom).

Phase	*a*	*b*	*c*	∆*H*	Ref.
V_5_C_3_(Cr_5_B_3_)	5.485	–	10.028	−0.371	This study
V_5_C_3_(W_5_Si_3_)	8.323	–	4.361	−0.188	This study
V_5_C_3_(Mn_5_Si_3_)	6.238	–	4.532	−0.265	This study
VC	4.156	–	–	−0.368	This study
VC	4.091	–	–	−0.405	Ref. 11
VC	4.158	–	–	−0.216	Ref. 8
V_2_C	4.540	5.726	5.031	−0.432	This study
V_2_C	4.495	5.628	4.929	−0.466	Ref. 11
V_2_C	4.551	5.735	5.032	−0.164	Ref. 8
V_4_C_3_	2.918	–	27.907	−0.421	This study
V_4_C_3_	2.948	–	27.782	−0.107	Ref. 8
V_6_C_5_	5.100	–	14.351	−0.503	This study
V_6_C_5_	5.005	–	14.099	−0.541	Ref. 11
V_6_C_5_	5.101	–	14.354	−0.052	Ref. 8
V_8_C_7_	8.326	–	–	−0.482	This study
V_8_C_7_	8.181	–	–	−0.522	Ref. 11
V_8_C_7_	8.329	–	–	−0.036	Ref. 8

**Table 2:** Calculated elastic constants *C*_ij_ (GPa), the minimum elastic eigenvalue *λ*_1_ (GPa), bulk modulus *B* (GPa), shear modulus *G* (GPa), Young’s modulus *E* (GPa),Poisson’s ratio *ν*, and hardness *H*_*ν*_ (GPa).

	*C*_11_	*C*_12_	*C*_13_	*C*_23_	*C*_22_	*C*_33_	*C*_44_	*C*_55_	*C*_6_	*B*	*G*	*E*	*ν*	*λ*_1_	*H*_*ν*_	Ref.
V_5_C_3_	539	179	193	–	–	492	193	–	153	278	179	441	0.235	153	21.85	This study
VC	668	130	–	–	–	–	198	–	–	309	224	542	0.208	198	29.54	This study
VC	748	139	–	–	–	–	182	–	–	342	224	551	0.230	–	25.90	Ref. 11
VC	663	122	–	–	–	–	203	–	–	302	228	546	0.198	–	31.48	Ref. 8
VC	578	147	–	–	–	–	176	–	–	291^c^	216^c^	519^c^	–	–	29.75	Ref. 9
V_2_C	393	181	122	189	381	410	107	125	131	240	118	303	0.290	107	7.73	This study
V_2_C	452	207	146	205	450	493	122	143	161	279	140	359	0.290	–	13.07	Ref. 11
V_2_C	400	182	120	189	383	414	110	130	135	242	121	311	0.286	–	11.70	Ref. 8
V_4_C_3_	512	124	137	–	–	477	99	–	194	253	148	372	0.255	113	16.87	This study
V_4_C_3_	537	154	206	–	–	480	148	–	–	299	162	412	0.271	–	16.15	Ref. 8
V_6_C_5_	452	108	130	–	–	472	190	–	172	235	176	422	0.200	154	26.36	This study
V_6_C_5_	505	126	155	–	–	512	229	–	190	266	198	475	0.200	–	28.23	Ref. 11
V_6_C_5_	456	114	130	–	–	474	189	–	–	237	176	423	0.202	–	26.07	Ref. 8
V_8_C_7_	528	107	–	–	–	–	162	–	–	247	180	432	0.207	162	25.81	This study
V_8_C_7_	651	120	–	–	–	–	179	–	–	297	210	509	0.210	–	27.44	Ref. 11
V_8_C_7_	512	108	–	–	–	–	167	–	–	243	180	433	0.203	–	26.37	Ref. 8

**Table 3:** Calculated elastic constants *C*_ij_ (GPa), the minimum elastic eigenvalue *λ*_1_ (GPa), bulk modulus *B* (GPa), shear modulus *G* (GPa), Young’s modulus *E* (GPa), Poisson’s ratio *ν* and hardness *H*_*ν*_ (GPa) of (V_1−*x*_Ti_*x*_)_5_C_3_.

x	C_11_	C_12_	C_13_	C_33_	C_44_	C_66_	B	G	E	ν	H_ν_	λ_1_
0.00	539	179	193	492	193	153	278	179	441	0.235	21.85	153
0.05	534	66	191	483	196	144	272	177	436	0.233	21.99	144
0.10	541	69	180	504	198	145	271	182	446	0.225	23.36	145
0.20	546	72	170	480	194	167	266	186	452	0.217	24.98	167
0.25	535	72	165	475	186	166	261	182	443	0.217	24.54	166
0.30	506	50	169	456	182	124	248	165	406	0.228	21.62	124
